# The diagnostic utility of diffusion weighted MRI imaging and ADC ratio to distinguish benign from malignant renal masses: sorting the kittens from the tigers

**DOI:** 10.1186/s12894-021-00832-5

**Published:** 2021-04-22

**Authors:** Suresh de Silva, Kathleen Rebecca Lockhart, Peter Aslan, Peter Nash, Anthony Hutton, David Malouf, Dominic Lee, Paul Cozzi, Fiona MacLean, James Thompson

**Affiliations:** 1 Faculty of Medicine, University of NSW, Kensington, NSW Australia; 2 Department of Radiology, I-MED Radiology Network, Sydney, Australia; 3 Department of Urology, St George Hospital, Kogarah, NSW Australia; 4 Hurstville Private Hospital, Hurstville, NSW Australia; 5 Department of Anatomical Pathology, Sonic Healthcare, Ryde, NSW Australia

**Keywords:** Renal neoplasms, Radiology, Urology, Magnetic resonance imaging

## Abstract

**Background:**

MRI is playing an increasing role in risk stratification and non-invasive diagnosis of the undifferentiated small renal mass. This study was designed to assess the reliability of MRI in diagnostic evaluation of renal masses, specifically characterising lesions with diffusion weighted imaging (DWI) and apparent diffusion coefficient (ADC) values.

**Methods:**

This is a retrospective analysis of patients undergoing MRI as part of their clinical workup for a renal mass suspicious for renal cell carcinoma (RCC) on CT or ultrasound followed by biopsy and/or surgical excision. All cases were conducted on 3 Tesla MRI, with conventional breath-held sequences, DWI and dynamic contrast enhanced phases. Tumour regions of interest were evaluated on ADC maps and compared with T2 weighted and post-contrast images.

**Results:**

Of the 66 renal tumours included, 33 (50.0%) were Clear Cell RCC, 11 (16.7%) were Oncocytoma, nine (13.6%) were Angiomyolipoma (AML), nine (13.6%) were Papillary RCC and four (6.1%) were Chromophobe RCC. Oncocytoma had the largest ADC values, significantly larger than AMLs and all RCC subtypes (*p* < 0.001). The average ADC value was also significantly larger in Clear Cell RCCs compared to AMLs, and other RCC subtypes (*p* < 0.001).

**Conclusions:**

MRI with DWI/ADC imaging may aid the differentiation of oncocytomas from RCCs and stratify RCC subtypes, Further studies are required to validate these findings.

*Trial registration*: Not applicable/retrospective study.

## Background

The incidental renal mass is a common diagnostic and management dilemma, since the widespread adoption of Computed Tomography (CT) and Ultrasound (US) in recent decades. Surgical resection is often considered the gold standard treatment as the majority are renal cell carcinomas (RCC). However, up to 33% of excised or biopsied renal masses have benign or indolent histopathology [1, 2]. If the nature of those masses could be reliably established by imaging then these patients could be spared the nephron loss, risks and costs of surgical intervention.

Conventional imaging cannot reliably differentiate benign or low-grade malignancies from high-grade malignancies. For example, benign oncocytomas, minimal fat angiomyolipomas (AMLs) and RCCs can have very similar CT and US characteristics [3]. Percutaneous biopsy aids pathological diagnosis, but risks include life-threatening bleeding, injury to surrounding organs, tumour seeding, sampling error underestimating overall grade and a significant non-diagnostic rate of 10–23% (often due to insufficient material) [1, 2]. Additionally, some masses are not amenable to biopsy (due to factors such as medial peri-hilar tumour location, predominately cystic composition, small size, single kidney, obesity or high bleeding risk).

MRI has developed an increasing role in non-invasive urologic tumour assessment (notably in prostate cancer), including the use of diffusion weighted imaging (DWI) with apparent diffusion coefficient values (ADC) [2–5]. DWI captures inherent differences between tissues in restricting water motion (Brownian motion) and ADC is a quantitative measure derived from DWI [6]. ADC is influenced by multiple factors including cellularity (validated in numerous studies, accounting for restricted diffusion in malignancy), cell membrane integrity, nuclear to cytoplasmic ratio and viscosity [7–9]. As renal tumour types vary with these structural properties, their ADC characteristics may allow for differentiation. There remains a paucity of published data in this area.

The aims of our study were to determine whether MRI ADC characteristics can be used to differentiate renal tumour types.

## Methods

This is a retrospective analysis of patients derived from a prospectively reported database of consecutive patients who underwent mpMRI for a de novo renal mass suspicious for RCC on CT or ultrasound as part of their clinical workup, followed by either percutaneous biopsy and/or surgical excision for histopathological diagnosis. All included cases were conducted on 3 Tesla (T) MRI, with improved signal-to-noise ratio/ spatial resolution compared to older 1.5 T MRI scanners. The scope of this study was only the select group for whom MRI was clinically indicated. The majority of local renal mass cases would not have had MRI or been excluded for a variety of reasons including but not limited to: pathognomonic findings on conventional imaging, offered to but declined by the patient due to personal preference for upfront biopsy/excision, symptoms requiring definitive treatment precluding need for further imaging, advanced stage of/metastatic cancer, a large tumour, contraindicated for MRI (e.g. non compatible medical implant or foreign body, significant chronic renal impairment, claustrophobia), the patient refusing to undergo subsequent biopsy/excision therefore the histopathological diagnosis, or a lesion ideally suited to partial nephrectomy in which MRI would be unlikely to change management.

Local health district ethical approval was obtained; individual patient consents were not sought as clinical practice was not changed in any way and all data was collected retrospectively and deidentified.

Between June 2014 and June 2019, all patients who had MRI imaging for renal masses at the same imaging facility were evaluated In total 75 renal masses with MRI imaging were included. Six were excluded due to non-standard imaging parameters or poor quality images that were not interpretable. Three masses were excluded due to (1) the presence of organising haematoma or (2) equivocal or unclassified diagnosis on histopathology. Five renal masses were diagnostic of Angiomyolipomas (AML), due to the presence of macroscopic fat without necrosis or calcification. These were included without tissue diagnosis to provide data for this benign tumour type; biopsy would have been unethical given their pathognomonic imaging findings and risk of bleeding with prominent vascularity. This left a final study cohort of 66 renal masses.

All patients underwent MRI on a 3T Siemens Skyra, using a 30 channel body array place over the pelvis and using the posterior coil elements of the in-table spine array. Scans included the following conventional non-contrast breath held sequences: Axial in and out of phase T1, Axial and Coronal 2D T2- weighted Haste, and Axial and Coronal 3D fat suppressed T1-weighted volume interpolated breathhold examination (VIBE) sequence. Dynamic contrast enhanced VIBE sequences obtained following the administration of gadolinium were included. The contrast agent (dose correlating to patient weight) was administered as an IV bolus using a power injector (Bracco) followed by a 30 ml saline flush, with both the contrast and saline injected at 2mls per second. Contrast enhanced sequences were obtained at cortico-medullary, nephrographic and excretory phases.

All scans included a DWI sequence performed in the axial plane before administration of contrast. DWI scans were a three- scan trace, monopolar with three diffusion directions. Diffusion b values were 50, 400 and 800, the DWI sequence used was 2D echoplanar, spin echo free breathing with a scan time of ~ 4:30 min. Fat Suppression was used, with a TR of 6100 ms and TE at min (~ 61 ms) ADC maps were obtained. Distortion correction was used with a noise level of 10. Slice thickness was 5 mm and 36 slices were obtained with a 38 cm FOV, EPI factor of 102 and a bandwidth of 1860 Hz/Px.

Imaging data was analysed by one sub-specialist radiologist with 15 years’ experience in evaluating body MRI imaging (SD). The ADC map of each renal mass was reviewed for the qualitative ADC value most representative of the tumour and a region of interest (ROI) then measured, expressed in units of mm^2^/second. The ADC maps were evaluated in conjunction with the T2 weighted and post contrast images to ensure placement of ROIs over solid tumour (not cystic/necrotic portions). A ROI of up to 50 mm^2^ was measured. In masses where qualitatively there were two ADC values which were approximately equally represented in the mass, two ROI were measured, one in each region measuring up to 50mm^2^. The combined value of these two regions averaged to obtain the ADC value.

Histopathology specimens (either biopsies, or excised specimens from partial or radical nephrectomy) were fixed in formalin, and processed in the usual manner. Microscopic examination was initially via hematoxylin and exosin stained slides, with immunoperoxidase and special stains utilized as required. The histology was diagnosed by six specialist uropathologists, and all cases were centrally reviewed by one highly experienced professor of uropathology specialising in renal histopathology (FM). All participating pathologists were blinded to the imaging results.

SPSS version 23 was used for analysis. To compare ADC values across the five lesion types investigated, analysis of variance (ANOVA) was used, accompanied by pairwise comparison of means undertaken using Tamhane’s T2 method, because ADC had been captured as a continuous variable measured on a ratio/interval scale while lesion type had been captured as a categorical variable with more than two (five) categories. Tamhane’s T2 method was chosen as the variance was likely to be significantly different between at least two tumour types compared. The 5% level of significance defined statistical significance.

The data analyses generated are available within the presented study but specific datasets are kept confidential due to their nature of including clinical and radiological details. Further information regarding dataset analysis may be available from the corresponding author upon request.

All methods were carried out in accordance with relevant guidelines and regulations, including those of the local health district and ethics committee.

## Results

**Fig. 1:**
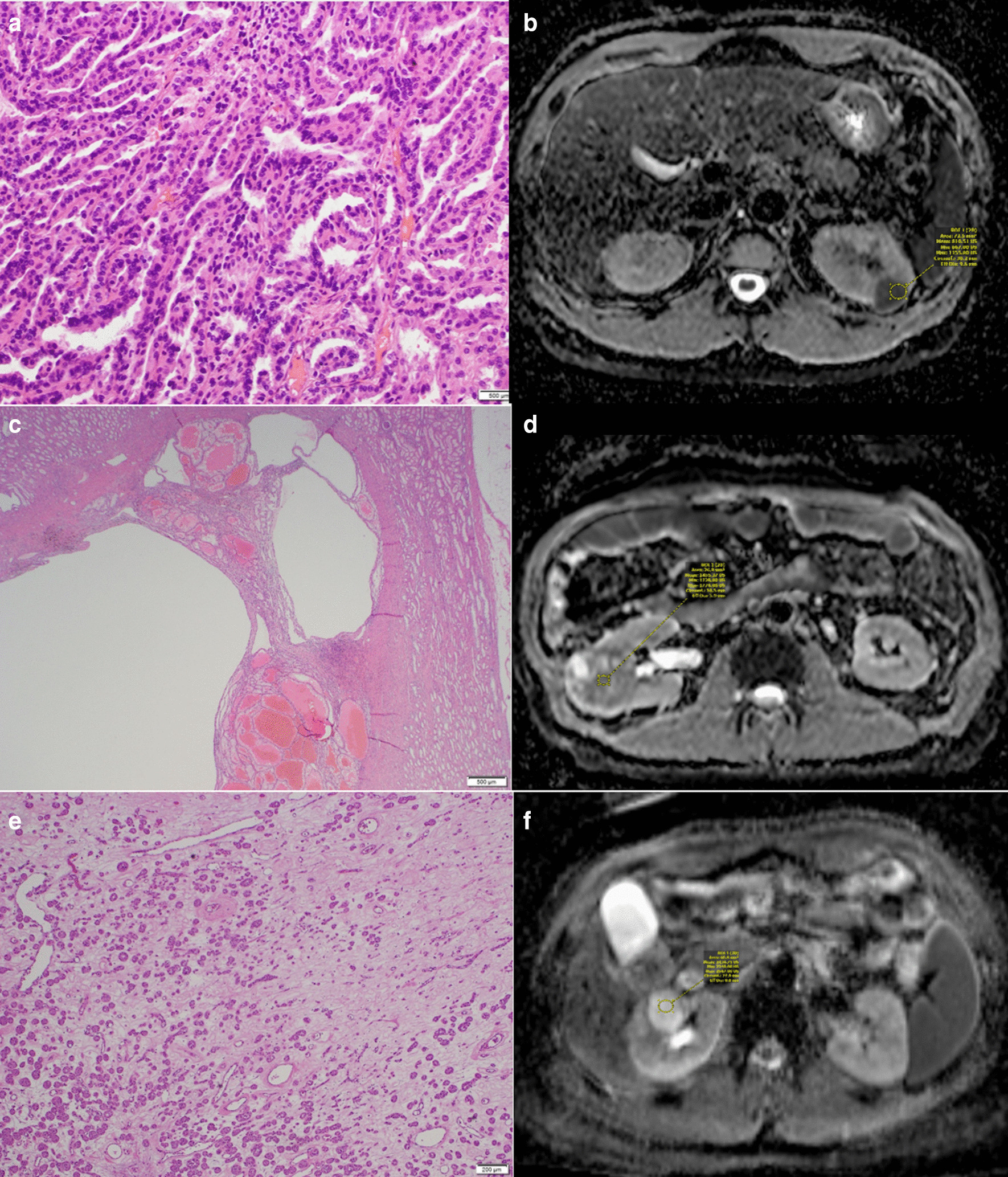
**a** Microscopy slide shows the cellular nature of papillary RCC which contributes to these tumours showing diffusion restriction and the low ADC number. **b** ADC map demonstrates a papillary RCC in the left kidney with low/ dark signal and an ADC number of only 0.81. **c** Microscopy slide shows the cystic change that can be seen in clear cell RCCs which contributes to them having a higher ADC number than non-clear cell RCCs. **d** ADC map shows low to intermediate signal in the clear cell RCC in the R Kidney, with the signal lower than that of Oncocytomas but higher than non-clear cell RCCs. **e** The microscopy slide demonstrates oedema, a feature of Oncocytomas, the key characteristic which produces the free diffusion and high ADC number. **f** ADC map demonstrates high signal intensity in an Oncocytoma in the right kidney with an ADC number of 2.43

Of the 66 patients with renal MRI examined for ADC characteristics, 33 (50.0%) were Clear Cell RCC, 11 (16.7%) were Oncocytoma, nine (13.6%) were Angiomyolipoma, nine (13.6%) were Papillary Type 1 RCC and four (6.1%) were Chromophobe RCC (Fig. 1). The renal masses had an average maximal dimension of 41 mm and a range of 9-126 mm.

**Fig. 2:**
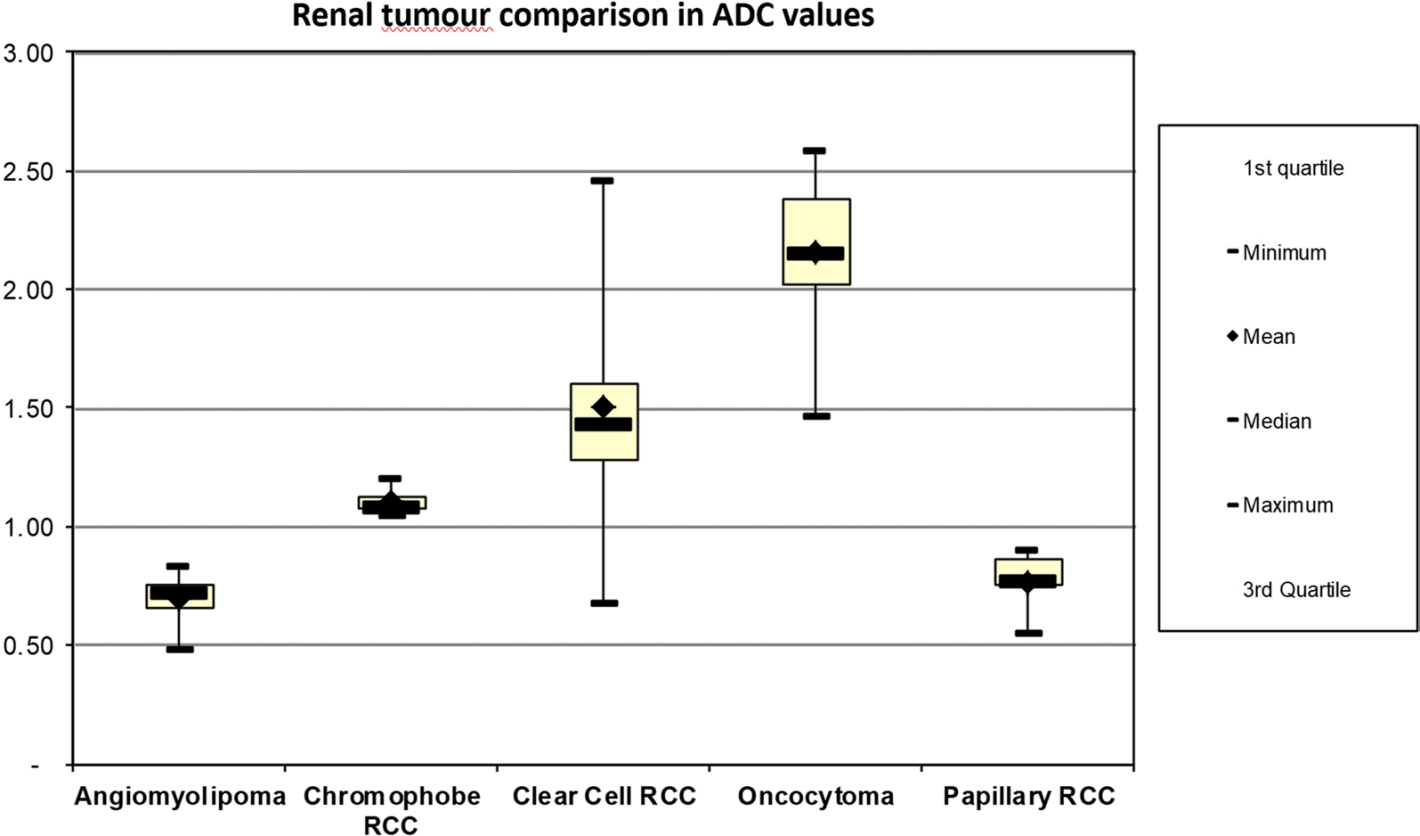
Comparison of ADC values between renal tumours

Oncocytoma had the largest ADC values, followed in descending order by Clear Cell RCC, Chromopohobe RCC, Papillary RCC and Angiomyolipoma. This is summarised in Fig. 2. In terms of histopathological gold standard diagnosis, 39.6% of masses were diagnosed with partial nephrectomy, 45.3% with radical nephrectomy and 15.1% with core biopsy. One patient had a non-diagnostic biopsy (this case was excluded from the analysis as described in Methods) and this patient had definitive successful focal ablation with cryotherapy.

ANOVA results indicated that the average ADC value difference was statistically significant for at least two tumour types (*p* < 0.001) with the assumptions of homogeneity of variance not being violated at the 1% level of significance (*p* > 0.01) (Table 1). Average ADC value was 2.1609 ± 0.2104 for Oncocytoma, 1.5033 ± 0.1328 for Clear Cell RCC, 1.1075 ± 0.1034 for Chromophobe RCC, 0.7611 ± 0.0942 for Papillary RCC and 0.6989 ± 0.0757 for AML (Table 1). At the 5% level of significance, the normality of distribution assumption was not violated (*p* > 0.05) (Fig. 2). Post-hoc analysis indicated that Oncocytoma average ADC value was significantly larger than that of AMLs (d = 1.46202, *p* < 0.001), Papillary (d = 1.39980, *p* < 0.001), Chromophobe (d = 1.05341, *p* < 0.001) and Clear Cell (d = 0.65758, *p* < 0.001) RCCs (Table 1). Average ADC value obtained was also significantly larger for Clear Cell RCC than for AML (d = 0.80444, *p* < 0.001), Papillary RCC (d = 0.74222, *p* < 0.001) and Chromophobe RCC (d = 0.39583, *p* < 0.001) (Table 1). Therefore, Oncocytoma can be differentiated from other tumours types investigated based on ADC values. Similarly, Clear Cell RCC can be differentiated from Angiomyolipoma, Papillary RCC and Chromophobe RCC. Although the box plots show some overlap, this is not the ultimate decision criteria as it is based on a sample, therefore significant p-values can still be obtained when there is overlap in box-plots. The ultimate decision criteria requires inferential testing/hypothesis testing where we have used the parametric ANOVA test and pairwise comparison. This enables the accurate assessment of the exact difference in groups with the results as noted above.

**Table 1:** Renal mass comparison of ADC value

	Clear Cell RCC	Angiomyolipoma	Papillary RCC	Chromophobe RCC	Oncocytoma
Mean	1.50	0.69	0.76	1.11	2.16
Standard error	0.07	0.04	0.04	0.03	0.09
Median	1.44	0.71	0.77	1.09	2.16
Mode	1	1	N/A	N/A	2
Standard deviation	0.37	0.10	0.12	0.06	0.31
Sample variance	0.14	0.01	0.02	0.00	0.10
Kurtosis	1.04	2.44	(0.31)	2.32	1.24
Skewness	0.74	(1.14)	(0.75)	1.41	(0.86)
Range	2	0	0	0	1
Minimum	1	0	1	1	1
Maximum	2	1	1	1	3
Sum	50	6	7	4	24
Count	33	8	9	4	11

## Discussion

Our results indicated that the average ADC of Oncocytomas was significantly higher than in all types of RCCs and AMLs, therefore able to be reliably identified and differentiated from other tumours. An ADC value of less than 1.9505 indicates the lesion is more likely to be an RCC or AML than an Oncocytoma. The addition of MRI in diagnostic workup could overcome some limitations of biopsy which is often unable to differentiate between oncocytoma and chromophobe RCC, and potentially avoid unnecessary surgery.

Other studies have found that Oncocytomas have significantly higher ADC values than clear cell RCCs but often within a similar range, making clarification between the two difficult even with MRI ADC mapping (although meta-analysis has supported the fact that ADC can differentiate between oncocytomas and malignant tumours in general) [10–13]. Our study, with the use of a 3T rather than 1.5 T MRI has demonstrated that oncocytomas and ccRCCs can be reliably discerned.

This study also demonstrates statistically significant differences in ADC value for clear cell RCCs and non-clear cell RCC subtypes. The average ADC value is significantly higher in clear cell RCC than papillary and chromophobe RCCs as well as AMLs. With an ADC measurement of less than 1.3705, the lesion is more likely to be a non-clear cell RCC of low malignant potential (chromophobe or type 1 papillary) than clear cell. This is consistent with the findings of other studies demonstrating the ADC difference between clear cell and non-clear cell RCC is statistically significant [10–12, 14].

Whereas most other groups have sampled/measured the darkest region on the ADC map we sampled the region most representative of the ADC in the mass. This may have improved diagnostic accuracy in this study. The technique we used to calculate the ADC value should be easily reproducible, and would lend itself to being part of a standardised protocol (although there is a general lack of protocol standardisation in renal MRI between imaging facilities currently). Renal MRI protocols are very much vendor specific and are influenced by several factors which include the strength of the magnet and the b values that are chosen to acquire the ADC calculations. These differences will contribute to variations in the ADC value of different tumour types between different studies. This can be mitigated by further studies using the same parameters with a consistent vendor as in this study.

MRI is likely to play an increasing role in renal tumour diagnosis as urologists are commonly faced with the management dilemma of an incidental renal mass where invasive investigation or surgical excision may carry high risk when compared to the option of active surveillance with imaging. For example, when biopsy is either non-diagnostic, not feasible due to anatomic factors (e.g. antero-medial or peri-hilar tumour location) or relatively contra-indicated due to patient factors (single kidney, anticoagulation with a high risk of thrombosis if temporarily withheld) [1, 15–17]. Surgical excision via partial or radical nephrectomy carries significant risk of major complications, quality of life effects and cost/ resource burdens on health systems [18, 19].

For these cases, performing an mpMRI with DWI/ADC may indicate likely tumour type and subtype/s. If the DWI/ADC strongly suggests a benign or indolent tumour such as an Oncocytoma or the urologist and patient may reach a shared decision for initial surveillance of the renal mass ifthe risks of invasive procedures outweigh the risks of surveillance. Conversely, in cases where the differential diagnosis includes aggressive malignancy (e.g. Clear Cell RCC), the risks of biopsy or excision are justified.

Our study has several limitations. As a retrospective study, it has the potential to introduce bias in measurement or selection. However, it represents a continuous cohort with clearly rationalised exclusions. Furthermore, all imaging and histopathology were reported prospectively and these assessors blinded. Although we demonstrated a statistically significant difference in ADC for non-clear cell RCCs, only 13 of these subtypes were included in this cohort (due to their relatively low prevalence), and further studies are required with larger population cohorts to validate these findings. Additionally, analysis of ADC measurements were all performed by a single experienced radiologist with an interest in kidney MRI, so further studies should assess for inter reader variability. The imaging completed in this study was with 3T MRI, which is not yet globally available (potentially affecting reproducibility) although it is higher quality imaging than 1.5 T MRI. Finally, a percentage of patients had biopsy diagnosis rather than excision, which has the potential of being less reliable in histopathological diagnosis.

In the evaluation of Renal MRIs, there is an urgent need to develop and validate a consensus -Ki-RADS MRI scanning and reporting system (including analyses such as ADC value) in order to guide radiologists and urologists in diagnostic probability.

## Conclusions

Our results suggest that renal MRI DWI/ADC imaging may aid the differentiation of oncocytomas and AMLs from RCCs and assist in stratifying RCC subtypes, especially in challenging cases where biopsy or surgery may be high risk or infeasible.

## Data Availability

The data analyses generated are available within the presented study but specific datasets are kept confidential due to their nature of including clinical and radiological details. Further information regarding dataset analysis may be available from the corresponding author upon request.

## Abbreviations

MRI: Magnetic resonance imaging
DWI: Diffusion weighted imaging
ADC: Apparent diffusion coefficient
RCC: Renal cell carcinoma
CT: Computed tomography
T: Tesla
ROI: Regions of interest
ANOVA: Analysis of variance
AML: Angiomyolipoma
US: Ultrasound
mpMRI: Multiparametric magnetic resonance imaging
VIBE: Volume interpolated breathhold examination
2D: Two-dimensional
TR: Repetition time
TE: Echo time
FOV: Field-of-view
EPI: Echo planar
Hz/Px: Hertz per pixel
CKD: Chronic kidney disease
